# Women with Lived Experience in the Perinatal Period: What do they want from Their Doctors?

**DOI:** 10.1192/j.eurpsy.2022.169

**Published:** 2022-09-01

**Authors:** C. Dolman, L. Howard, I. Jones

**Affiliations:** 1 Institute of Psychiatry, Psychology & Neuroscience at King’s College London, Health Service And Population Research Department, Womens Mental Health, AF, United Kingdom; 2 University of Cardiff, Wales, Mrc Centre For Neuropsychiatric Genetics And Genomics, Cardiff, United Kingdom

**Keywords:** Perinatal, Women with SMI, Optimum care, lived experience

## Abstract

**Introduction:**

Best practice requires the treating physician to understand the needs and hopes of his/her patient, particularly in relation to pregnancy and childbirth preferences. This is even more necessary for women with Severe Mental Illness (SMI) because of the complicated decisions they face balancing the need to continue medication in pregnancy to prevent relapse against any possible harm to the foetus. Objectives: To explore what women themselves view as most important when discussing pregnancy and childbirth with psychiatrists and what barriers there are to a) having a meaningful conversation and b) achieving optimum outcomes. Qualitative methods were used to analyse the data from in-depth interviews with 21 women, recruited from a South London NHS organisation (76%) and the UK’s national bipolar charity (24%). The views of 25 health professionals, including 19 psychiatrists, were also collected and analysed. Results: Many themes emerged but principally women wanted: information, continuity of care, better training for health professionals, to co-produce a detailed care plan, access to a Mother and Baby Unit, peer support and more research on medications in pregnancy. Conclusions: This study highlighted the importance of understanding women’s needs and fears and giving them the necessary information to make the difficult decisions that face them. Such understanding is likely to lead to more positive therapeutic relationships and better long-term outcomes.

**Disclosure:**

No significant relationships.

